# Improvement of dicarboxylic acid production with *Methylorubrum extorquens* by reduction of product reuptake

**DOI:** 10.1007/s00253-022-12161-0

**Published:** 2022-09-15

**Authors:** Laura Pöschel, Elisabeth Gehr, Markus Buchhaupt

**Affiliations:** 1 DECHEMA-Forschungsinstitut, Microbial Biotechnology, Theodor-Heuss-Allee 25, 60486 Frankfurt am Main, Germany; 2 Department of Life Sciences of the Goethe University Frankfurt am Main, Max-von-Laue-Str. 9, 60438 Frankfurt am Main, Germany

**Keywords:** Dicarboxylic acids, *Methylorubrum extorquens*, Product reuptake, Acid transporters, Ethylmalonyl-CoA pathway, Methylotroph

## Abstract

**Abstract:**

The methylotrophic bacterium *Methylorubrum extorquens* AM1 has the potential to become a platform organism for methanol-driven biotechnology. Its ethylmalonyl-CoA pathway (EMCP) is essential during growth on C1 compounds and harbors several CoA-activated dicarboxylic acids. Those acids could serve as precursor molecules for various polymers. In the past, two dicarboxylic acid products, namely mesaconic acid and 2-methylsuccinic acid, were successfully produced with heterologous thioesterase YciA from *Escherichia coli*, but the yield was reduced by product reuptake. In our study, we conducted extensive research on the uptake mechanism of those dicarboxylic acid products. By using 2,2-difluorosuccinic acid as a selection agent, we isolated a dicarboxylic acid import mutant. Analysis of the genome of this strain revealed a deletion in gene *dctA2*, which probably encodes an acid transporter. By testing additional single, double, and triple deletions, we were able to rule out the involvement of the two other DctA transporter homologs and the ketoglutarate transporter KgtP. Uptake of 2-methylsuccinic acid was significantly reduced in *dctA2* mutants, while the uptake of mesaconic acid was completely prevented. Moreover, we demonstrated *M. extorquens*-based synthesis of citramalic acid and a further 1.4-fold increase in product yield using a transport-deficient strain. This work represents an important step towards the development of robust *M. extorquens* AM1 production strains for dicarboxylic acids.

**Key points:**

• *2,2-Difluorosuccinic acid is used to select for dicarboxylic acid uptake mutations.*

• *Deletion of dctA2 leads to reduction of dicarboxylic acid uptake.*

• *Transporter-deficient strains show improved production of citramalic acid.*

**Supplementary Information:**

The online version contains supplementary material available at 10.1007/s00253-022-12161-0.

## Introduction

Given the limited availability and global concern about the sustainability of fossil resources, the development of sustainable production processes for prevalent chemicals is indispensable. Currently, a new field of biotechnological processes is emerging that focuses on the use of alternative carbon sources. To be sustainable, these carbon sources must be derivable from renewable sources and must not compete with food production. Methanol, whose production share from renewable sources and waste streams starts to increase (Roode-Gutzmer et al. 2019), offers itself as an alternative raw material for biotechnological processes of the future. Moreover, its use as feedstock minimizes the risk of contamination during biotechnological applications and reduces the cost of downstream processing by using a defined minimal medium.

**Fig. 1:**
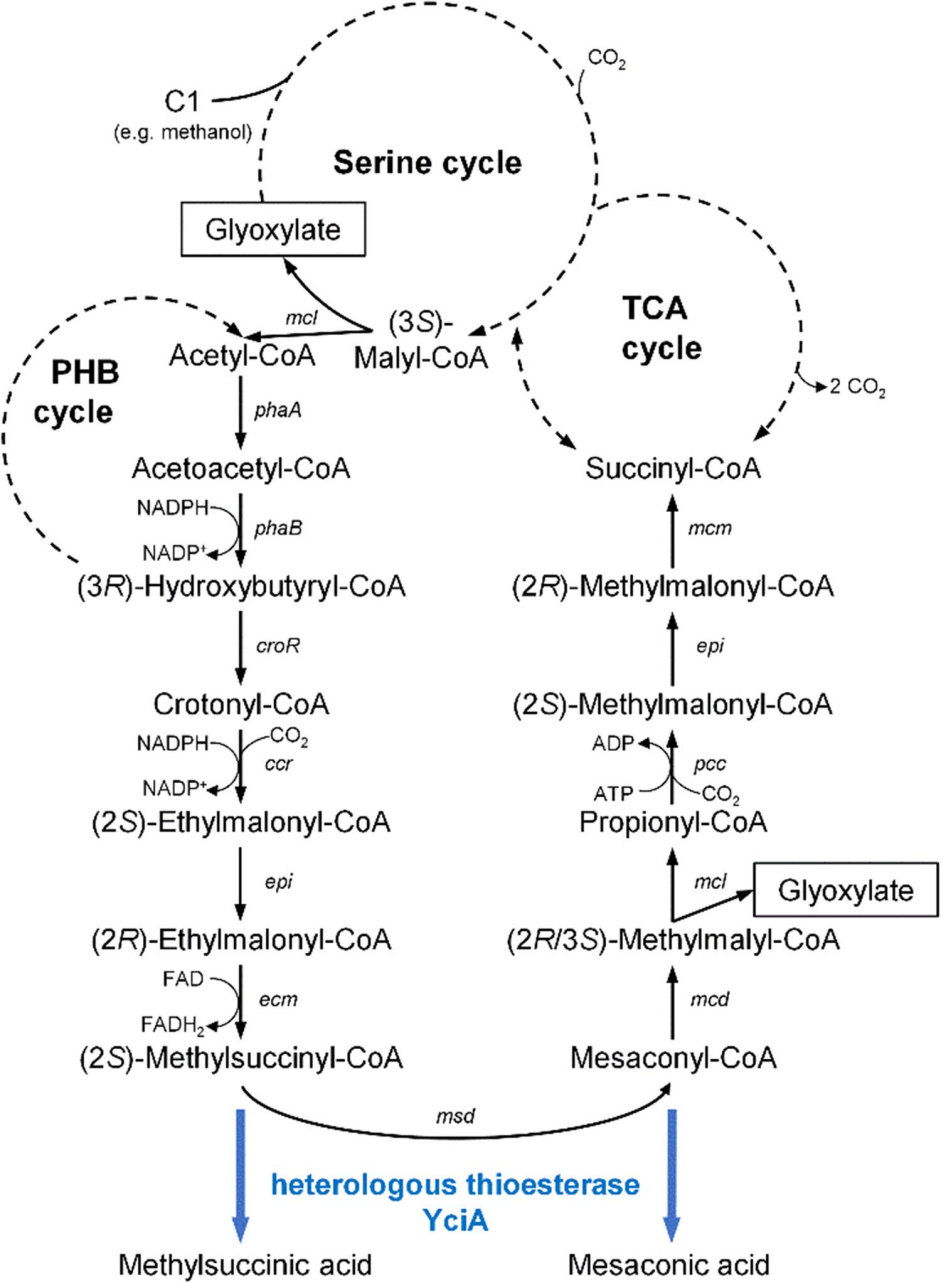
Overview of ethylmalonyl-CoA pathway (EMCP) *in M. extorquens* AM1. The EMCP is interlaced with the serine cycle, the PHB cycle, and the tricarboxylic acid cycle. Cleavage of (2*R*/3*S*)-methylmalyl-CoA by malyl-CoA/beta-methylmalyl-CoA lyase (*mcl*) releases glyoxylate, which replenishes the serine cycle (indicated by boxes). Further genes of EMCP are: β-ketothiolase (*phaA*); acetoacetyl-CoA reductase (*phaB*); crotonase (*croR*); crotonyl-CoA carboxylase/reductase (*ccr*); ethylmalonyl-CoA epimerase (*epi*); ethylmalonyl-CoA mutase (*ecm*); methylsuccinyl-CoA dehydrogenase (*msd*); mesaconyl-CoA dehydratase (*mcd*); propionyl-CoA carboxylase (*pcc*); methylmalonyl-CoA mutase (*mcm*). Expression of heterologous thioesterase-encoding gene *yciA* leads to hydrolysis of (2*S*)-methylsuccinyl-CoA and mesaconyl-CoA (bold blue arrows). The corresponding products, namely 2-methylsuccinic acid and mesaconic acid, are released into the supernatant

A group of widely used chemicals that are currently mainly produced from fossil raw materials are polyamides and polyesters. Biotechnologically produced dicarboxylic acids can serve as sustainable platform chemicals for the production of those polymers (Jang et al. 2012). Although pathways for microbiological production of dicarboxylic acids from classical feedstocks have been known for many years (Lee et al. 2011; Alonso et al. 2014), methanol-based routes for carboxylic acid production have been described only recently for the methylotrophic bacterium *Methylorubrum extorquens* AM1 (DSM 1338), e.g., for itaconic acid, 3-hydroxypropionic acid, mesaconic acid, and 2-methylsuccinic acid (Sonntag et al. 2014; Yang et al. 2017; Lim et al. 2019). The latter two are directly derived from the ethylmalonyl-CoA pathway (EMCP) by enzymatic hydrolysis (Fig. 1). The EMCP as an anaplerotic pathway is essential for methylotrophic growth of *M. extorquens*. It regenerates glyoxylate, which is needed for replenishment of the serine cycle and includes two CO_2_-fixing steps (Erb et al. 2007; Peyraud et al. 2009). The pathway is also essential for the assimilation of C2 compounds during growth on e.g. acetate in microorganisms having no glyoxylate pathway as *M. extorquens* (Erb et al. 2010; Okubo et al. 2010). The EMCP consists of 11 enzymes and includes several CoA-bound dicarboxylic acid intermediates with high biotechnological potential (Alber 2011). Mixtures of mesaconic acid and 2-methylsuccinic acid were produced from respective CoA-precursors by introducing the thioesterase YciA originating from *Escherichia* *coli* (Sonntag et al. 2014). The high carbon flux via the EMCP enabled remarkable product titers to be achieved without further modification of metabolic pathways. In both studies of Sonntag and colleagues (2014; 2015), the concentration of dicarboxylic acid products in supernatant was reduced remarkably (i.e., 45% or 60% over a period of 20 h, respectively) as soon as the cells were reaching the stationary growth phase. This behavior is probably attributable to product reuptake. An adjustment of the cobalt concentration in the growth medium from 12.6 µM to 0.2 µM did not only lead to a sixfold increase in product yield due to biomass formation-limiting inhibition of certain EMCP enzymes, but also decreased the reuptake of products in stationary growth phase (Sonntag et al. 2015). Despite the lack of obvious product reuptake under these low-cobalt conditions in the late cultivation phase, it remains unclear whether product uptake continuously proceeds during the acid production phase. This would resemble a partial futile cycle, leading to loss of resources and therefore decreased product yield. The phenomenon of product reuptake was also described for 3-hydroxypropionic acid, another EMCP-derived product from *M. extorquens* AM1, even when using a low cobalt concentration of 1.26 µM in the medium (Yang et al. 2017). These observations make it worth to investigate the product uptake mechanism and to prevent it on a molecular level.

The main targets for prevention of product uptake are the dicarboxylic acid import proteins. It has been observed that a 30-fold reduction in sodium concentration in the growth medium resulted in ceasing of product uptake (Sonntag et al. 2015), suggesting that carriers of the DctA family may be involved, which are known to be sodium-dependent (Janausch et al. 2002). A transposon mutant study by Van Dien and coworkers identified a mutant with a disrupted *dctA* homolog that was no longer able to use succinate as sole carbon source (Van Dien et al. 2003). The affected protein was referred to as GenBank entry A33597. Since this mutant strain was promising for dicarboxylic acid production, the gene encoding the protein with the highest similarity to A33597 was deleted by Sonntag et al. (2015). Surprisingly, the strain did not show the phenotype previously described. Sonntag et al. (2015) speculated that the deleted gene might not be identical to the gene mutated in the Van Dien study, as the GenBank entry A33597 in question represents a *Sinorhizobium meliloti* protein sequence and therefore cannot be unambiguously assigned.

Another possibility of preventing the reuptake of products is to convert them to non-metabolizable molecules. For example, the hydration product of mesaconic acid, namely (*S*)-citramalic acid, would not only fulfill this criterion, but would also extend the product spectrum of *M. extorquens* AM1 by a chiral, enantiopure product. The mesaconase/fumarate hydratase from *Paraburkholderia xenovorans* (Kronen et al. 2015) seems promising for heterologous expression for this purpose.

In our study, we aimed at reducing reuptake of dicarboxylic acids at the molecular level. Using a simple and straightforward selection approach, we identified a mutant (partial deletion of *dctA2*) with reduced uptake of mesaconic acid and 2-methylsuccinic acid. We confirmed our results by construction of deletion mutants and were able to rule out the involvement of other DctA transporters. In addition, we have successfully implemented the production of a new dicarboxylic acid product from *M. extorquens* AM1, whose production also benefits from the transport deficiency. These new insights into dicarboxylic acid transport contribute to the development of *M. extorquens* AM1 towards a comprehensive production platform for methanol-based biotechnology.

## Material and methods

### Bacterial strains and growth conditions

*Escherichia coli* DH5α (Hanahan 1985; Grant et al. 1990) cultures were grown in LB medium (Bertani 1951) at 37 °C. For cultivation of *M. extorquens* AM1 (DSM 1338, Peel and Quayle 1961), minimal medium was prepared as described before (Peyraud et al. 2009), containing 123 mM methanol, 31 mM sodium succinate or 5 mM sodium acetate as carbon source, respectively. CoCl_2_ concentration in the medium was set to 12.6 µM (Kiefer et al. 2009; Sonntag et al. 2014). For solid medium, agar–agar at 15 g/L was added. For cultivation of *M. extorquens* AM1 in liquid medium, 5 mL precultures in methanol minimal medium were grown in test tubes for 48 h at 30 °C and 180 rpm on a rotary shaker. Main cultures of 20 mL were subsequently inoculated to an OD_600_ of 0.1 in 100 mL shake flasks and incubated under the same conditions and in the same medium as the precultures. If required, tetracycline-hydrochloride at 10 μg/mL, kanamycin sulfate at 30 μg/mL (for *E. coli*), or kanamycin sulfate at 50 μg/mL (for *M. extorquens* AM1) was added to the medium. For high-resolution measurements of growth curves, 1 ml of main cultures were incubated in a BioLector® microbioreactor system in 48-well Flowerplates® (m2p-labs GmbH, Baesweiler, Germany) at 30 °C and 1000 rpm. For complementation experiments, glyoxylate was added to a final concentration of 370 mg/L (5 mM). Depending on the specific production experiment, heterologously expressed genes encoding thioesterase YciA from either *E. coli* (YciAEc) or *H. influenza* (YciAHI) and a gene encoding a fumarate hydratase (mesaconase) from *Paraburkholderia xenovorans* (MesaPx) was used. The two thioesterases produce similar amounts of products with slightly different ratios. For YciAHI, a crystal structure is available (3BJK, Willis et al. 2008).

### Chemicals

All chemicals were purchased from Carl Roth (Karlsruhe, Germany), VWR International (Darmstadt, Germany), or Merck (Darmstadt, Germany). Solvents for chromatography were purchased in LC–MS grade quality.

### Genome sequencing

DNA of *M. extorquens* AM1 wild type and mutant strains was sequenced by Illumina sequencing (MiSeq; 2 × 250 bp; 1–2 million PE-Reads; GenXPro, Frankfurt, Germany). Paired and cleaned Illumina reads were trimmed with BBDuk Trimmer (JGI, Berkeley, USA) to a quality cut-off of 20. Mapping to the reference genome (NCBI NC_012807–NC_012811, annotation date 04/11/2021) and calling for SNPs was done with Geneious Prime (Biomatters, Auckland, New Zealand). Assembling qualities and base coverages are listed in Online Resource Table S1.

### DNA cloning and plasmid construction

All standard plasmid cloning procedures were performed in *E. coli* DH5α. Plasmid DNA was purified with GeneJET Plasmid Miniprep Kit from Thermo Scientific (Waltham, USA). Polymerase chain reactions (PCR) were performed with Q5 Polymerase from New England Biolabs (Frankfurt, Germany) according to the manufacturer’s protocol. Subsequently, PCR products were purified using the DNA Clean & Concentrator Kit from Zymo Research Europe (Freiburg, Germany). Oligonucleotides were purchased from Merck (Darmstadt, Germany), restriction enzymes and T4 ligase from NEB. All genetic constructs were confirmed by Sanger sequencing at Eurofins Scientific (Luxembourg, Luxembourg). Transformation of *M. extorquens* AM1 was done as described before (Toyama et al. 1998). Genomic DNA of *M. extorquens* AM1 was purified with GenElute™ Bacterial Genomic DNA Kit from Merck (Darmstadt, Germany).

### Generation of dicarboxylic acid transporter deletion mutants

Knockout of the genes encoding potential carboxylic acid importers (*dctA1*: MEXAM1_RS15430, *dctA2*: MEXAM1_RS10985 and *dctA3*: MEXAM1_RS20450) and the ketoglutarate permease KgtP (*kgtP*: MEXAM1_RS24315) were carried out with allelic exchange vector pCM184 carrying a kan^R^ antibiotic resistance cassette (Marx and Lidstrom 2002). Vector pCM184_∆*dctA1* was constructed as described in the publication by Sonntag et al. (2015), in which *dctA1* is referred to as *dctA*. The deletion vectors pCM184_∆*dctA2*, pCM184_∆*dctA3* and pCM184_∆*kgtP* were designed accordingly in two constitutive cloning steps. First, 500 bp of genomic upstream flanking region of the respective gene of interest was amplified while introducing restriction sites at both ends of the PCR product. The resulting fragment was digested with according restriction enzymes and ligated to equally digested pCM184 (Table 1). The resulting vectors carrying the upstream fragment were subsequently treated in the same manner to introduce the previously digested downstream fragment to result in the final allelic exchange vectors. After restreaking *M. extorquens* AM1 transformants on solid methanol medium containing kanamycin, the correct integration of resistance marker was verified with respective primer pairs, binding inside and outside of the cassette. Screening for single recombination mutants and removal of the resistance marker with *cre*-expression vector pCM157 was achieved on solid methanol medium containing tetracycline as described by Marx and Lidstrom (2002). Elimination of pCM157 was performed by cultivating strains overnight in medium without antibiotics and screening single colonies for tetracycline sensitivity. Deletion of the complete ORF was double checked by PCR of genomic DNA. All used oligonucleotides are listed in Table 1. For multiple gene deletions, the described procedure was performed several times in succession. Single, double and triple deletion mutants were simultaneously created in *M. extorquens* AM1 wild type as well as in the ∆*cel* strain (Delaney et al. 2013).

**Table 1:** Oligonucleotides, plasmids, and strains used in this work. Capital letters in DNA sequences indicate restriction sites. Restriction enzymes used for subcloning into pCM184 are indicated in brackets. Optimization of RBS sequences was done with RBS Calculator 2.1 (Salis 2011)

Name	Sequence/genotype	Description/application	Reference
Bacterial strains			
* E. coli* DH5α	F^–^ φ80*lac*ZΔM15 Δ(*lac*ZYA-*arg*F)U169 *rec*A1 *end*A1 *hsd*R17(r_K_^–^, m_K_^+^) *pho*A *sup*E44 λ^–^*thi*-1 *gyr*A96 *rel*A1	Standard cloning applications	Hanahan (1985); Grant et al. (1990)
* M. extorquens* AM1	Cm^R^, Gram-negative, facultative methylotrophic, obligate aerobic, α-proteobacterium		Peel and Quayle (1961)
* M. extorquens* AM1 DFS mutant 1	*M. extorquens* AM1 with chromosomal 12 bp deletion within MEXAM1_RS10985 (*dctA2*)	Mutants isolated by selection with DFS	This work
* M. extorquens* AM1 DFS mutant 2	*M. extorquens* AM1 with chromosomal mutation of MEXAM1_RS18205 (SLC13 family transporter gene)		This work
Wild type ∆*dctA3*	*M. extorquens* AM1 with chromosomal deletion of MEXAM1_RS20450	Dicarboxylic acid transporter deletion strains	This work
Wild type ∆*dctA1* ∆*dctA3*	*M. extorquens* AM1 with chromosomal deletion of MEXAM1_RS15430 and MEXAM1_RS20450		This work
Wild type ∆*dctA1* ∆*dctA2* ∆*dctA3*	*M. extorquens* AM1 with chromosomal deletion of MEXAM1_RS15430, MEXAM1_RS10985 and MEXAM1_RS20450		This work
* M. extorquens* AM1 ∆*cel* (CM2720)	*M. extorquens* AM1 with chromosomal deletion of cellulose biosynthesis genes	Reduced biofilm formation without loss of fitness	Delaney et al. (2013)
∆*cel* ∆*dctA1*	*M. extorquens* AM1 ∆*cel* with chromosomal deletion of MEXAM1_RS15430	Dicarboxylic acid transporter deletion strains in ∆*cel* strain background	This work
∆*cel* ∆*dctA2*	*M. extorquens* AM1 ∆*cel* with chromosomal deletion of MEXAM1_RS10985		This work
∆*cel* ∆*kgtP*	*M. extorquens* AM1 ∆*cel* with chromosomal deletion of MEXAM1_RS24315		This work
∆*cel* ∆*dctA1* ∆*dctA2*	*M. extorquens* AM1 ∆*cel* with chromosomal deletion of MEXAM1_RS15430 and MEXAM1_RS10985		This work
∆*cel* ∆*dctA2* ∆*dctA3*	*M. extorquens* AM1 ∆*cel* with chromosomal deletion of MEXAM1_RS10985 and MEXAM1_RS20450		This work
∆*cel* ∆*dctA1* ∆*dctA2* ∆*dctA3*	*M. extorquens* AM1 ∆*cel* with chromosomal deletion of MEXAM1_RS15430, MEXAM1_RS10985 and MEXAM1_RS20450		This work
Plasmids			
pCM184	Kan^R^, Tc^R^, Amp^R^ oriT, pBR322ori	Allelic exchange vector for gene deletion in *M. extorquens*	Marx and Lidstrom (2002)
pCM157	Tc^R^, oriT, pBR322ori	Cre recombinase expression plasmid	Marx and Lidstrom (2002)
pCM184_Δ*dctA1*	pCM184 containing ~ 500 bp flanking sites of *dctA1*	Allelic exchange vector for *dctA1*	Sonntag et al. (2015)
pCM184_Δ*dctA2*	pCM184 containing ~ 500 bp flanking sites of *dctA2*	Allelic exchange vector for *dctA2*	This work
pCM184_Δ*dctA3*	pCM184 containing ~ 500 bp flanking sites of *dctA3*	Allelic exchange vector for *dctA3*	This work
pCM184_Δ*kgtP*	pCM184 containing ~ 500 bp flanking sites of *kgtP*	Allelic exchange vector for *kgtP*	This work
pCM160	Kan^R^, pmxaF, oriT, pBR322ori	Constitutive expression vector for *M. extorquens*	(Marx and Lidstrom 2001)
pCM160_RBS_*yciA*Ec	pCM160 containing codon-optimized thioesterase gene *yciA* from *E. coli*, optimized RBS	Constitutive expression of *yciA*Ec	Sonntag et al. (2014)
pCM160_RBS_*yciA*HI	pCM160 containing codon-optimized thioesterase gene *yciA* from *Haemophilus influenzae*, optimized RBS	Constitutive expression of *yciA*HI	This work (GenBank ON109394)
pCM160_RBS_*yciA*Ec_MesaPx	pCM160 containing codon-optimized thioesterase gene *yciA* from *E. coli*, codon-optimized fumarate hydratase gene *bxe_A3136* from *P. xenovorans*, optimized RBS	Constitutive expression of *yciA*Ec *and* fumarate hydratase/ mesaconase (WP_038456612, (Kronen et al. 2015))	this work (GenBank ON109395)
Oligonucleotides			
dctA1_up_fw	actaGACGTCagcggaagcgaactctgcg (AatII)	Amplification of up- and downstream fragments	Sonntag et al. (2015)
dctA1_up_rev	actaCATATGgggcgtttctccctgtcgga (NdeI)		
dctA1_down_fw	actaTACGTAtccggtcaggagggcgcac (SnaBI)		
dctA1_down_rev	actaGAGCTCagggcttcgggcgtatcgag (SacI)		
dctA2_up_fw	ccGACGTCtcggtctggtcgctcatgg (AatII)		This work
dctA2_up_rev	actaCCATGGtcctcaccgataccgtgttgc (NcoI)		This work
dctA2_down_fw	actaTACGTAatgagcctgccaccgctc (SnaBI)		This work
dctA2_down_rev	actaGAGCTCaaccgcgatgcccgaacc (SacI)		This work
dctA3_up_fw	ccGACGTCtgcacgctccacgagaagc (AatII)		This work
dctA3_up_rev	ggaattcCATATGcaccgccgtttagggcgaat (NdeI)		This work
dctA3_down_fw	actaTACGTAaacgggcttccgcttcgg (SnaBI)		This work
dctA3_down_rev	actaGAGCTCaacaccgccaccgagtacg (SacI)		This work
kgtP_up_fw	actaCCATGGactcacatccacaacgcgc (NcoI)		This work
kgtP_up_rev	actaGCGGCCGCcgctgggacgcaagacga (NotI)		This work
kgtP_down_fw	actaCCGCGGtgagcccttatcagaggccg (SacII)		This work
kgtP_down_rev	actaTACGTAacatcagaattgcggccgtc (SnaBI)		This work
kan_up_dctA1_check_fw	aactcgatcttcacgacgac	Verification of integration site of kanamycin resistance marker	This work
kan_up_dctA2_check_fw	tttcgtagaggccgatgtc		This work
kan_up_dctA3_check_fw	ttgaaggcctcgttatgc		This work
kan_up_kgtP_check_fw	agatcgaagtgttcgacctc		This work
kan_up_check_rev	agacgtttcccgttgaatatg		Sonntag et al. (2015)
kan_down_check_fw	agtttcatttgatgctcgatgag		
kan_down_dctA1_check_rev	atacagcttggtatcaaccg		This work
kan_down_dctA2_check_rev	tcgaggatgttgaccacc		This work
kan_down_dctA3_check_rev	ctccttcttcaagaccgac		This work
kan_down_kgtP_check_rev	cacgatctcgtaggtgtc		This work
check_del_dctA1_fw	agccatgactgaactgcag	Verification of complete gene deletion	This work
check_del_dctA1_rev	aatcgcgaagcagcaatg		This work
check_del_dctA2_fw	aatacgcggctaggtcg		This work
check_del_dctA2_rev	tcttgatcaggccggtg		This work
check_del_dctA3_fw	atcaatgccgtacccgc		This work
check_del_dctA3_rev	gaccggatgggtctaagga		This work
check_del_kgtP_fw	tctcgaaggagcggctc		This work
check_del_kgtP_rev	tctccggcatctgtcatgg		This work

### Dicarboxylic acid analysis

For quantification of dicarboxylic acid products, the supernatant of *M. extorquens* AM1 cultures was centrifuged for 5 min at 16.000 g and passed through a 0.22 μm PDVF-syringe filter (Carl Roth, Karlsruhe, Germany). For the quantification of mesaconic acid and 2-methylsuccinic acid, 10 µL of the cell free supernatant was chromatographed on a 150 × 4.6 mm Rezex™ ROA-Organic Acid H + (8%) column (Phenomenex, Aschaffenburg, Germany) at 30 °C oven temperature in a SLC10-A HPLC system (Shimadzu, Duisburg, Germany). 5 mM H_2_SO_4_ in MilliQ water was used as mobile phase. Samples were analyzed with a SPD20A UV–Vis detector at 205 nm as described in Sonntag et al. (2014). Retention times of the analytes are listed in Table 2. For the quantification of samples containing additional dicarboxylic acid products (e.g., citramalic acid), which all have similar retention times, an alternative LC–MS/MS setup was used. Chromatography of 1 µL of the respective samples was done on a 150 × 4.6 mm Luna Omega 3 µm PS C18 100 Å column (Phenomenex, Aschaffenburg, Germany) in a Nexera X2 UHPLC system (Shimadzu, Duisburg, Germany). Separation was performed isocratically at 12% [v/v] acetonitrile and 88% [v/v] ddH_2_O, both containing 0.2% [v/v] formic acid at 40 °C oven temperature. The column was washed after every run by raising the acetonitrile content in the mobile phase to 95%. Mesaconic acid, 2-methylsuccinic acid, citramalic acid as well as other potential EMCP-derived carboxylic acid products (Table 2) were analyzed on a LCMS-8045 system (Shimadzu). The analytes were negatively ionized with APCI or ESI ion source, fragmented and finally quantified by comparing the results to calibration curves of corresponding standards. Quantification was performed with the LabSolutions software (Shimadzu). The retention times and the manufacturers of the standard substances are listed in Table 2. Fragmentation of (2*S*,3*R*)-2-hydroxy-3-methylsuccinic acid and (*S*)-citramalic acid for unambiguous identification is shown in Online Resource Fig. S1.

**Table 2:** Analytical standards and retention times used for the quantification of carboxylic acids produced by *M. extorquens* AM1

Standard substance	Manufacturer	Retention times [min] in HPLC measurements	Retention times [min] in LC–MS/MS measurements
Crotonic acid	Carl Roth (Karlsruhe, Germany)	n.d	1.06
(2*S*,3*R*)-2-Hydroxy-3-methylsuccinic acid	Enamine (Riga, Latvia)	n.d	1.08
(*S*)-Citramalic acid	Merck (Darmstadt, Germany)	n.d	1.08
Ethylmalonic acid		n.d	1.58
Mesaconic acid		12.88	1.38
2-Methylsuccinic acid		8.10	1.32
Methylmalonic acid		n.d	1.20
Succinic acid		n.d	1.02

### Transcriptome analysis

A whole transcriptome analysis was done for *M. extorquens* AM1 harboring pCM160_RBS_*yciAHI* or pCM160, respectively. Samples were taken after 22.75, 31.75, 47, 51.25 and 70.5 h of cultivation. For stabilization of RNA, one volume of growing bacterial culture (> 10^6^ cells) was mixed with two volumes of RNAprotect Bacteria Reagent from QIAGEN (Hilden, Germany) and incubated for 5 min. Cells were centrifuged for 10 min at 5000 g to remove supernatant and stored pelleted at − 80 °C. RNA was isolated and analyzed by GenXPro (Frankfurt, Germany) via Illumina sequencing (2–5 million reads; 2 × 75 bp). Raw read counts were normalized by CPM (counts per million) method to compensate for varying sample sequencing depth. For graphical representation, transcript levels were scaled to the count of the respective gene in the control strain at the first sampling point. The transcriptome dataset was deposited at NCBI (accession number GSE199961).

## Results

### Search for dicarboxylic acid transporter candidates by transcriptome analysis

**Fig. 2:**
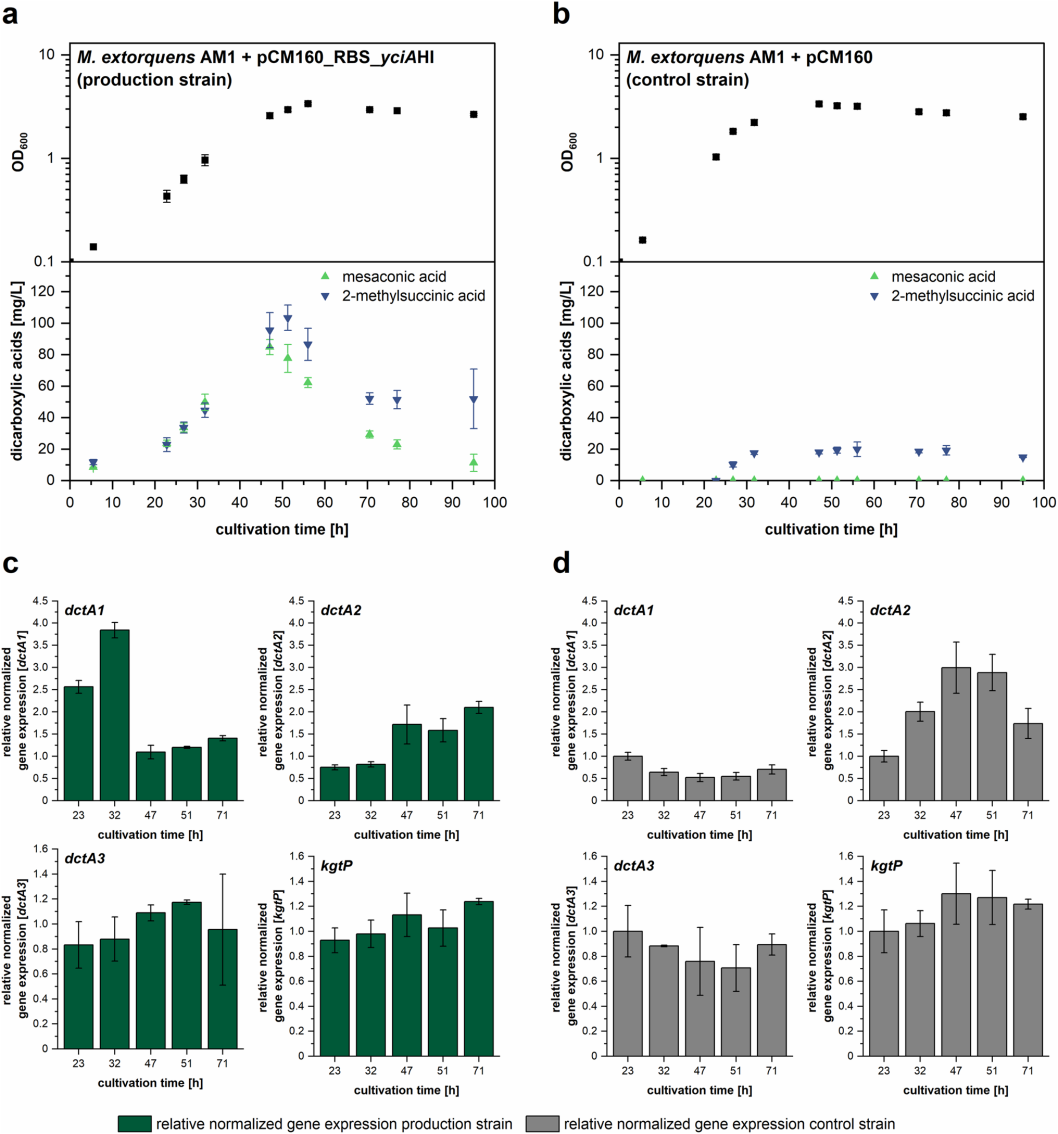
Transcriptome analysis of dicarboxylic acid production strain to identify candidates for product uptake factors. **a**–**b** Growth kinetics and time-dependent concentration of mesaconic acid and 2-methylsuccinic acid in supernatant of *M. extorquens* AM1 + pCM160_RBS_*yciAHI* (**a**) or *M. extorquens* AM1 + pCM160 (**b**) growing in methanol minimal medium. Other EMCP-derived carboxylic acid products with titers insufficient for quantification (< 5 mg/L) are not displayed. **c** Normalized relative gene expression of *dctA1*, *dctA2*, *dctA3*, and *kgtP* for *M. extorquens* AM1 + pCM160_RBS_*yciAHI*. **d** Normalized relative gene expression of *dctA1*, *dctA2*, *dctA3*, and *kgtP* for *M. extorquens* AM1 + pCM160. The transcript levels are scaled to the transcript count of the respective gene in the control strain *M. extorquens* AM1 + pCM160 at 23 h of cultivation. Error bars represent standard deviations from three independent replicates. An additional visualization of the data in form of products per OD_600_ can be found in Online Resource Fig. S2

*M. extorquens* AM1 naturally produces small amounts of dicarboxylic acids derived from the EMCP pathway. Sonntag and colleagues succeeded in increasing the amounts of released 2-methylsuccinic acid and mesaconic acid to a combined titer of 0.13 g/L by expressing the thioesterase encoding gene *yciA* from *E. coli* heterologously in a methanol minimal medium containing 12.6 µM CoCl_2_ (Sonntag et al. 2014, 2015). However, at the end of the exponential growth phase, the acid concentrations started to decrease again, which is probably caused by product-reuptake and metabolization. It is unclear whether product uptake also occurs during the exponential growth phase, which could reduce the overall yield. To address this question, we investigated the dicarboxylic acid importers of *M. extorquens* AM1. A number of candidates with homology to the multispecies dicarboxylic acid transporter DctA (Janausch et al. 2002) were identified by BLAST analysis by Sonntag et al. (2014). In the referred study (Sonntag et al. 2014), unexpectedly and in contrast to other publications (Van Dien et al. 2003), a *dctA* knockout mutant (MEXAM1_RS15430) was still able to grow on succinate. Therefore, it was questioned whether *dctA* (hereinafter referred to as *dctA1*) from both studies was in fact the identical ORF. Sonntag et al. also identified two other *dctA* homologs (MEXAM1_RS10985 and MEXAM1_RS20450, referred to as *dctA2* and *dctA3* in our study) and an additional dicarboxylic acid transporter encoding gene (*kgtP* MEXAM1_RS24315, Seol and Shatkin 1991) in the genome sequence, but these were not further investigated (Sonntag et al. 2014). To monitor expression levels of the homolog candidates and to potentially identify additional dicarboxylic acid importers in our study, *M. extorquens* AM1 cells expressing an alternative thioesterase gene from *Haemophilus influenzae* (*yciA*HI) were analyzed on a whole transcriptome level together with an empty vector control strain. Additionally, product titers were determined. Small amounts of the EMCP derived carboxylic acids (2*S*,3*R*)-2-hydroxy-3-methylsuccinic acid, crotonic acid, ethylmalonic acid, methylmalonic acid and succinic acid were detected, but their titers were irrelevant and insufficient for quantification (< 5 mg/L, also present in the empty vector control culture). *M. extorquens* AM1 cells harboring plasmid pCM160_RBS_*yciA*HI released up to 104 ± 8 mg/L 2-methylsuccinic acid and up to 85 ± 9 mg/L mesaconic acid in the production phase, followed by clear decreases of 2-methylsuccinic acid and mesaconic acid titers (Fig. 2a).

The relative expression levels of dicarboxylic acid transporter candidates identified by Sonntag and colleagues (2014) were analyzed in the production strain as well as in cells harboring an empty control vector. Hydrolysis of EMCP-esters by a heterologous thioesterase is a strong intervention in the primary carbon metabolism of the cell. This is reflected by the different growth kinetics of production and control strain (Fig. 2a,b). Therefore, we decided not to perform an analysis of differential gene expression. Instead, relative gene expression was determined for each strain separately, before comparing the kinetic patterns of both strains. The gene *dctA1*, which has been already deleted in the work of Sonntag et al. (2015), showed a higher relative gene expression rate compared to control strains (Fig. 2c,d). This effect is particularly noticeable in the early sampling points, whereas expression was comparably strong during the main acid production phase (e.g., equivalent timepoints 47 h in production strains vs. 23 h or 32 h in control strains). Furthermore, moderately increased expression levels at later time points in the production strain were observed for *dctA2*, *dctA3*, and *kgtP* but at least for *dctA2* and *kgtP* a similar pattern was found in the non-producing reference strain.

We could not identify any additional genes with acid transporter annotation within the transcriptome data that were significantly higher expressed in cells sampled at the late stages of cultivation (51 h or 71 h) compared to the earlier sampling time points in the data set. Therefore, the transcriptome analysis of *M. extorquens* AM1 harboring pCM160_RBS_*yciA*HI yielded no candidates for potential dicarboxylic acid uptake factors.

### Investigation of 2,2-difluorosuccinic acid as potential selection agent for dicarboxylic acid transport mutants

**Fig. 3:**
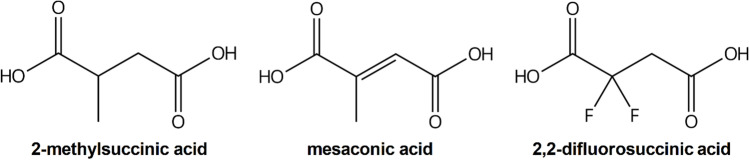
Chemical structures of 2-methylsuccinic acid, mesaconic acid, and 2,2-difluorosuccinic acid

Since the transcriptome analysis did not provide clear hints for genes with clear upregulation before the phase of product reuptake, a more direct approach was considered to identify cells incapable of dicarboxylic acid uptake. For selection for respective *M. extorquens* cells with an uptake defect, 2,2-difluorosuccinic acid (DFS), a presumably cytotoxic dicarboxylic acid with structural similarity to the target products, was used (Fig. 3).

**Fig. 4:**
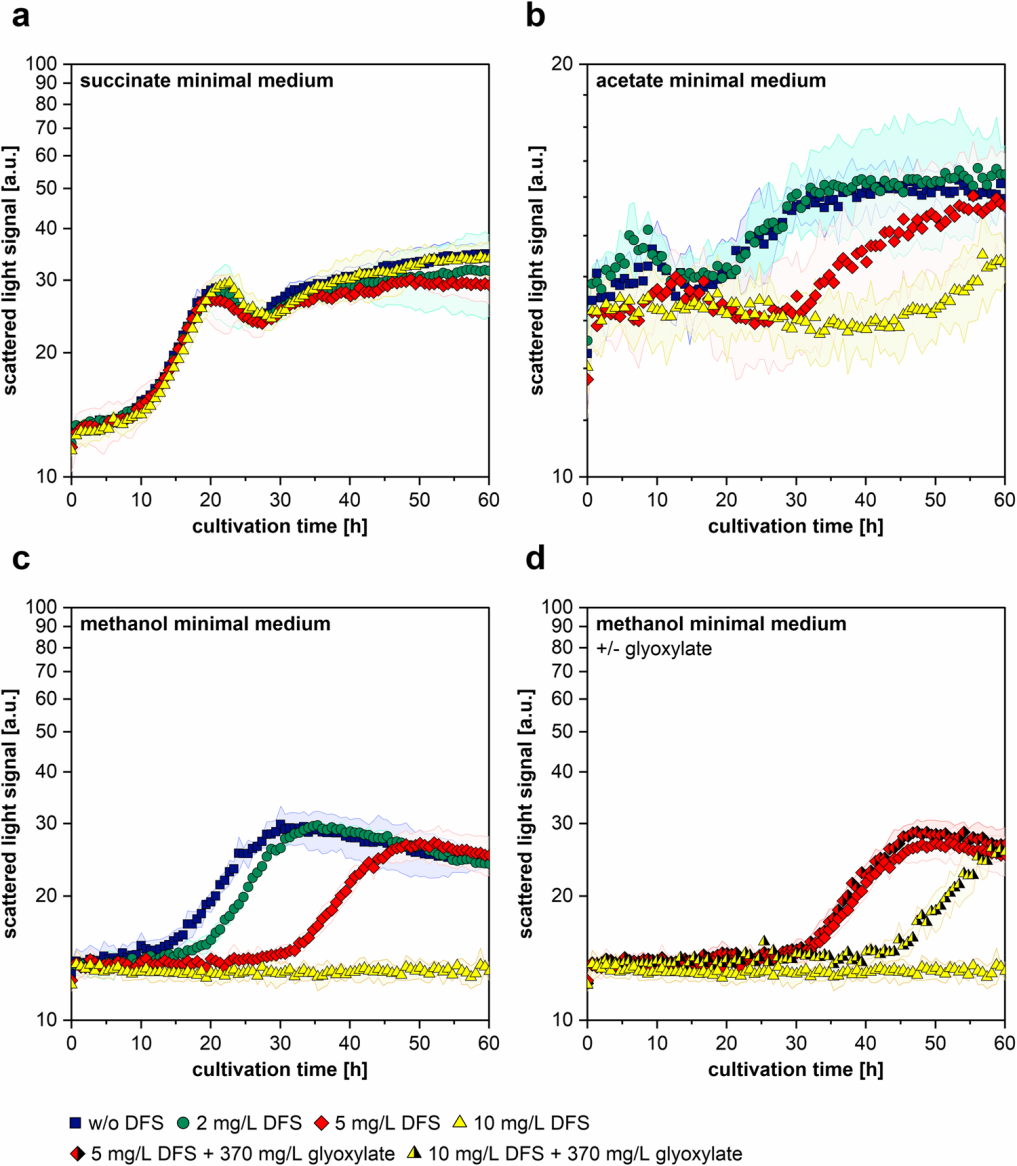
Investigation of toxic effects of DFS on *M. extorquens* AM1. **a**–**c** Growth of *M. extorquens* AM1 in succinate, acetate, or methanol minimal medium with different additives. Cultures were grown in a microbioreactor. Growth was monitored by scattered light signal. 2,2-Difluorosuccinic acid (DFS) was added at the start of cultivation in concentrations of 2 mg/L, 5 mg/L, or 10 mg/L. *M. extorquens* AM1 culture without DFS was used as a negative control. For better readability, figure part **b** is scaled differently. **d** Complementation experiment, in which 370 mg/L of glyoxylate was added to cultures containing 5 mg/L or 10 mg/L of DFS, respectively. Colored areas represent standard deviations from three independent replicates

To investigate the effect of DFS, the growth of *M. extorquens* AM1 in minimal medium containing different carbon sources and different concentrations of DFS was monitored in a microbioreactor system. Although the differences between replicates growing on succinate medium were quite high, the results showed, that the addition of 2, 5, or 10 mg/L DFS had no significant effect on growth (Fig. 4a). In contrast, the addition of DFS to acetate or methanol containing cultures resulted in suppression of growth and prolonged lag phases (Fig. 4b,c). The addition of 2 mg/L DFS to the methanol medium resulted only in a slight delay in growth. In turn, the addition of 5 mg/L resulted in a greater delay and a reduction of the maximum cell density, and the addition of 10 mg/L DFS completely inhibited growth (Fig. 4c). Although maximum cell densities were lower, similar effects were observed when cells were cultured in acetate medium (Fig. 4b). Since an active EMCP in *M. extorquens* AM1 is required for the use of methanol or acetate as sole carbon source (Peyraud et al. 2009), we assumed that DFS might target the EMCP. This assumption was reinforced by the fact, that the DFS-mediated growth defect in methanol medium could be partially compensated by the addition of glyoxylate (Fig. 4d). The provision of glyoxylate is the essential function of the EMCP during growth on C1 or C2 compounds (Fig. 1, Peyraud et al. 2009) and the rescue of EMCP mutants by glyoxylate addition was already demonstrated many years ago (Salem and Quayle 1971; Chistoserdova and Lidstrom 1996).

**Fig. 5:**
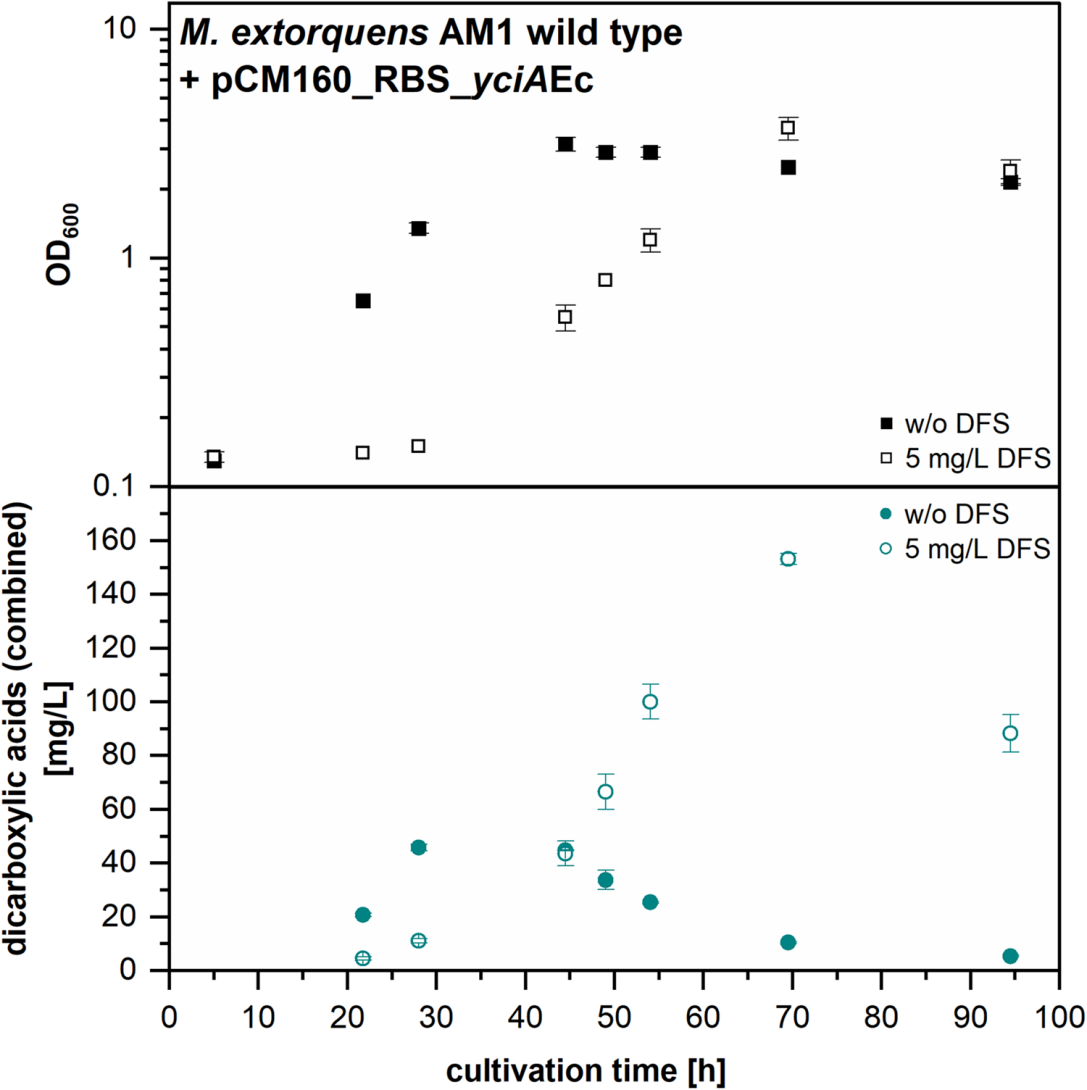
Growth kinetics and time-dependent combined concentration of dicarboxylic acid products (mesaconic acid and 2-methylsuccinic acid) in supernatant of *M. extorquens* AM1 harboring pCM160_RBS_*yciA*Ec in methanol minimal medium (*filled symbols*) and in methanol minimal medium with addition of 5 mg/L 2,2-difluorosuccinic acid (DFS) after 5 h of cultivation (*empty symbols*). Other EMCP-derived carboxylic acid products with titers insufficient for quantification (< 5 mg/L) are not displayed. Error bars represent standard deviations from two independent replicates. An additional visualization of the data in form of products per OD_600_ can be found in Online Resource Fig. S3

When DFS was added to an *M. extorquens* AM1 culture expressing the *E. coli yciA* gene, we observed a growth delay but at the same time the production of 2-methylsuccinic acid and mesaconic acid was raised to a maximum measured combined product titer of 153 mg/L ± 2 mg/L (Fig. 5). Even though the measured time points can only give an indication of the complete production kinetics, the product titers were clearly increased compared to the control experiment without DFS addition. This experiment provided further indication of an EMCP inhibitory effect, as EMCP flux limitation leads to increased dicarboxylic acid production in thioesterase expression strains (Sonntag et al. 2015).

Altogether, our investigations showed DFS to be a suitable selection agent for dicarboxylic acid uptake mutants. However, the mutant selection has to be performed under conditions, which require a functional EMCP, as this pathway seems to contain the molecular target for the compound.

### Identification of potential dicarboxylic acid uptake transporters by using DFS

**Fig. 6:**
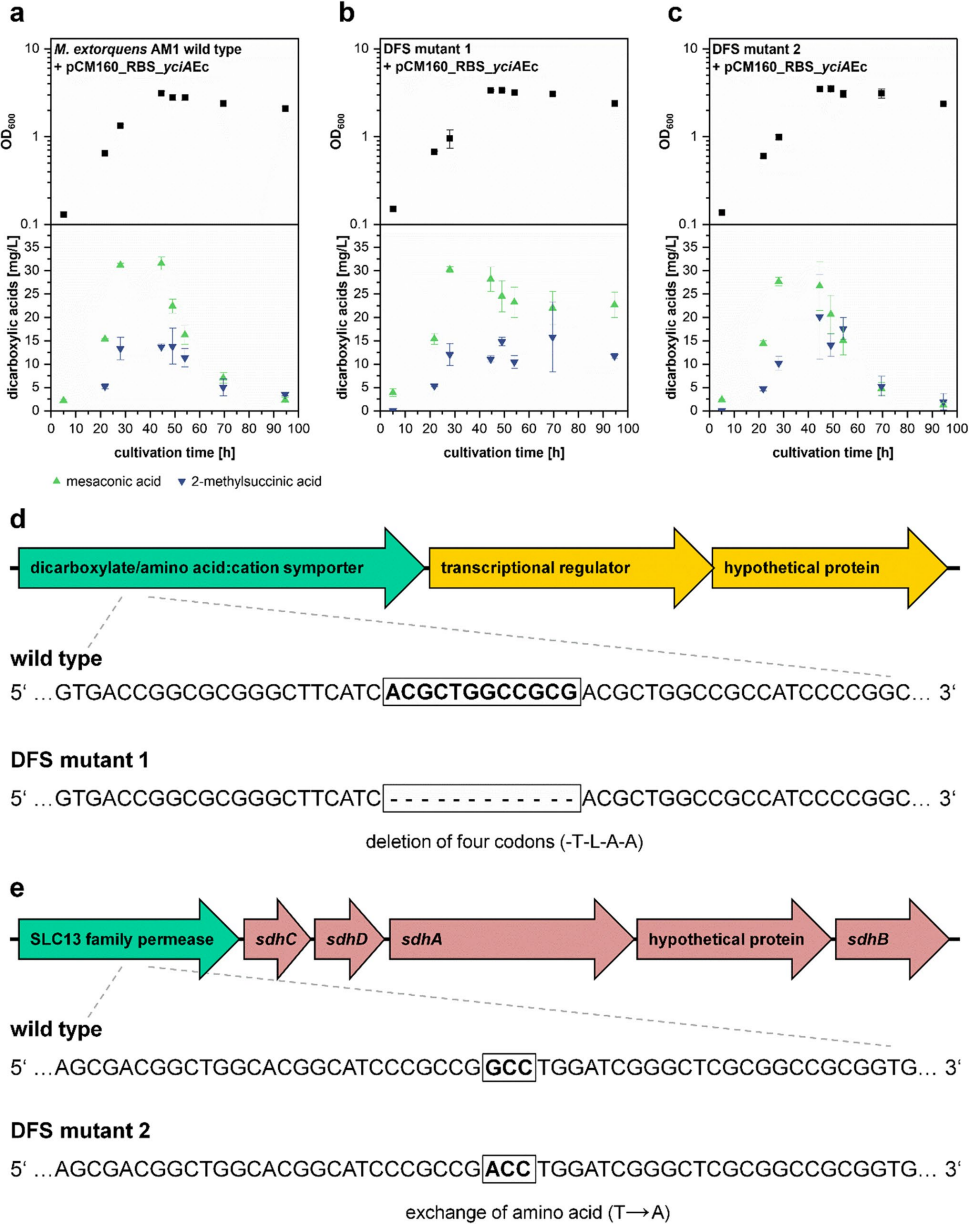
Phenotypes and genotypes of DFS-resistant mutants. **a**–**c** Growth kinetics and time-dependent concentration of mesaconic acid and 2-methylsuccinic acid in supernatant of *M. extorquens* AM1 wild type + pCM160_RBS_*yciA*Ec (**a**), DFS mutant 1 + pCM160_RBS_*yciA*Ec (**b**), and DFS mutant 2 + pCM160_RBS_*yciA*Ec (**c**), growing in methanol minimal medium. Other EMCP-derived carboxylic acid products with titers insufficient for quantification (< 5 mg/L) are not displayed. Error bars represent standard deviations from three independent replicates. **d** Gene locus region of MEXAM1_RS10985-MEXAM1_RS1097, in which DFS mutant 1 has a 12 bp deletion within the dicarboxylic acid transporter MEXAM1_RS10985 (≙ *dctA2*). **e** Gene locus region MEXAM1_RS18205-MEXAM1_RS18230, in which a mutation in a SLC13 family permease encoding gene could be identified in the genome of DFS mutant 2. An additional visualization of the data in form of products per OD_600_ can be found in Online Resource Fig. S4

To isolate DFS-resistant strains, an *M. extorquens* AM1 culture was transferred to solid minimal medium containing acetate or methanol as carbon source and additional 5 mg/L DFS. After 18 days of incubation, a small number of colonies could be observed (one colony on methanol medium, 20 colonies on acetate medium). One strain from each medium was isolated. Restreaking the cells on solid succinate, methanol, or acetate minimal medium resulted in wild-type-like growth behavior. Isolated *M. extorquens* DFS mutant 1 (isolated from acetate-DFS minimal medium) and DFS mutant 2 (isolated on methanol-DFS minimal medium) were transformed with plasmid pCM160_RBS_*yciA*Ec and tested for dicarboxylic acid production along with an *M. extorquens* AM1 wild type strain also containing plasmid pCM160_RBS_*yciA*Ec. None of the strains showed a growth defect in liquid methanol minimal medium (Fig. 6a-c). Although a slight decrease in mesaconic acid concentration was still observed in the culture of DFS mutant 1 after 40 h of cultivation, it showed significantly reduced product reuptake compared to the wild type control strain. DFS mutant 2 showed production/uptake kinetics similar to the wild type control. The genomes of the wild type control strain, DFS mutant 1 and DFS mutant 2 were sequenced to identify any new mutations. A total of 11 identical mutations were found in all three strains, which differ from the NCBI reference genome sequence (Online Resource Table S2). In DFS mutant 1, an additional 12 bp deletion within the dicarboxylic acid transporter gene *dctA2* (MEXAM1_RS10985) could be identified (Fig. 6d). Although the *dctA2* gene product is probably involved in product reuptake, a residual uptake was still observed in DFS mutant 1. In DFS mutant 2, a single point mutation was identified causing an amino acid exchange in a SLC13 family permease (MEXAM1_RS18205, Fig. 6e). This mutation was probably responsible for the DFS resistance phenotype. Nevertheless, the strain did not show a decreased reuptake of mesaconic acid and 2-methylsuccinic acid behavior (Fig. 6c) observed for DFS mutant 1.

### Mesaconic and 2-methylsuccinic acid production behavior of strains lacking one or several transporter-encoding genes

**Fig. 7:**
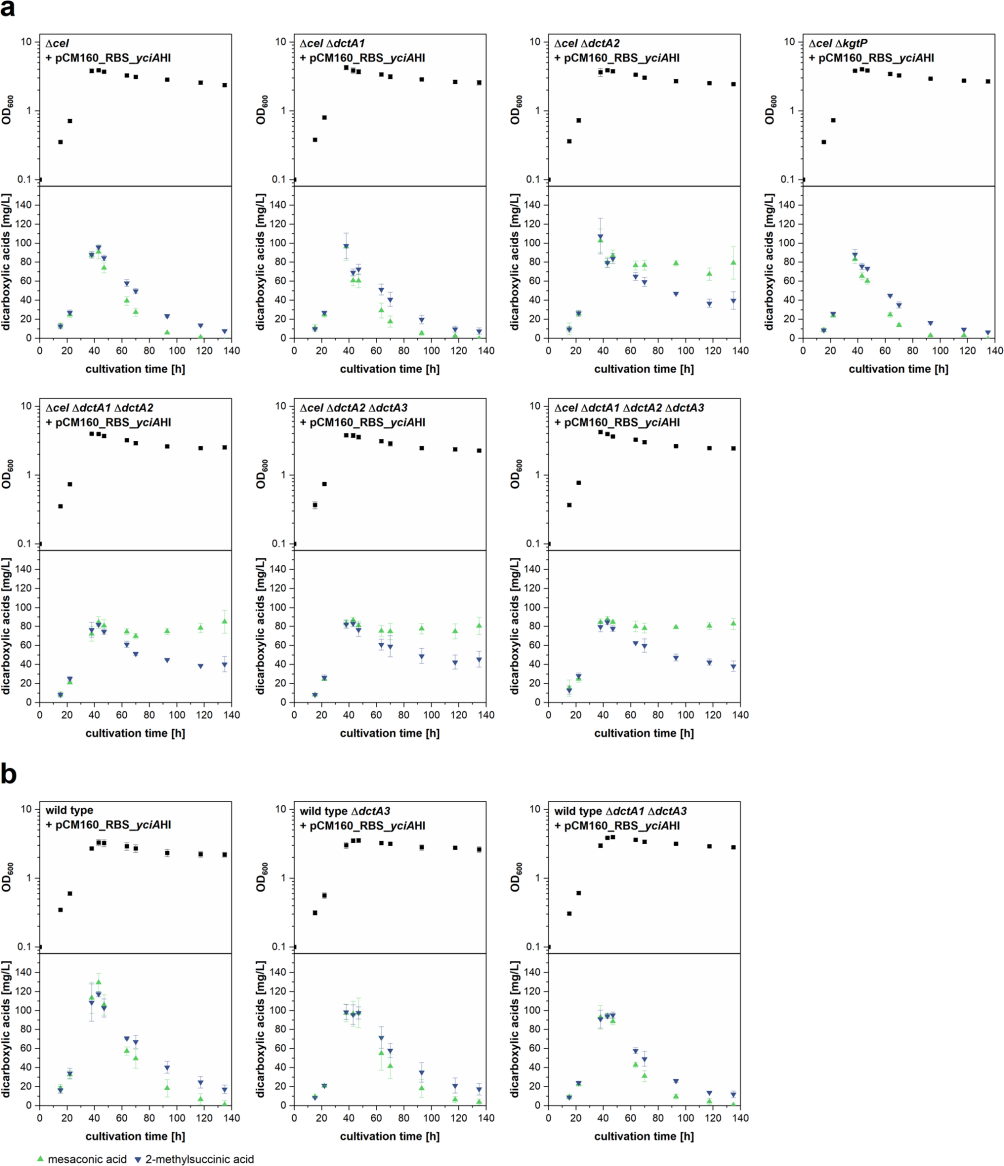
Growth kinetics and time-dependent concentration of mesaconic acid and 2-methylsuccinic acid in supernatant of *M. extorquens* AM1 cells without and with single, double or triple transporter deletions. Strains heterologously express thioesterase encoding gene *yciA*HI in methanol minimal medium. Other EMCP-derived carboxylic acid products with titers insufficient for quantification (< 5 mg/L) are not displayed. Error bars represent standard deviations from three independent replicates. Strains were constructed based on either **a** *M. extorquens* AM1 wild type or **b** *M. extorquens* AM1 ∆*cel* strain. An additional visualization of the data in form of products per OD_600_ can be found in Online Resource Fig. S5

The 12 bp deletion in the *dctA2* gene in DFS mutant 1 conferred a reduced reuptake of mesaconic acid and 2-methylsuccinic acid, yet the reuptake was only partially prevented. This could indicate that the transporter protein variant lacking four amino acids is still partially active or that more than one import protein is involved in the reuptake of the products. Aiming at complete suppression of reuptake, we constructed single, double and triple deletion strains lacking one, two or three putative dicarboxylic acid transporter genes (start to stop codon). Besides *dctA2,* this deletion approach involved also *dctA1* and *dctA3*. Additionally, a ∆*kgtP* knockout strain was constructed and analyzed. To also obtain strains with high process suitability, we introduced the deletions not only in *M. extorquens* AM1, but also in *M. extorquens* AM1 ∆*cel*. Since the latter lacks the cellulose synthesis operon, it has been shown to be more suitable for downstream applications as biofilm formation and cell clumping are reduced (Delaney et al. 2013). Although we attempted to achieve a triple *dctA* deletion by testing different sequences of consecutive gene deletions, not all combinations could be achieved in both, the wild type and the ∆*cel* strain. Nevertheless, all possible combinations could be achieved in one of the two strains. Since production behavior did not differ significantly between both strains, the phenotypes of all combinations of transporter gene deletions could be analyzed. Since we observed that YciAHI resulted in higher product titers compared to YciAEc (e.g., Fig. 2 versus Fig. 5) and a crystal structure for YciAHI is available that may facilitate further developments (3BJK, Willis et al. 2008), the YciAHI thioesterase was chosen over the *E. coli* enzyme for the experiment. It was introduced into all constructed transporter deletion strains to investigate the effects on product reuptake during shake flask cultivations with methanol minimal medium. Single deletions of *dctA1*, *dctA3*, or *kgtP* had no effect on the production or reuptake behavior of mesaconic acid or 2-methylsuccinic acid compared to the reference strains (Fig. 7a,b). Only the effect caused by complete deletion of the *dctA2* gene is clearly evident and it is comparable to the effect caused by the 12 bp deletion in the *dctA2* gene in DFS mutant 1. The same applies to strains with double or triple deletions: Only in strains containing at least the *dctA2* deletion, a reduced dicarboxylic acid uptake could be observed after cell growth stopped, whereas additional deletions did not lead to additional phenotypes. In all ∆*dctA2* strains, product levels increased during the exponential growth phase of the cells as in the control strains. The product concentrations per biomass during the growth phase and therefore also the maximum product levels determined at the end of the exponential growth phase were, however, not higher than those observed in the production strain without transporter gene deletions (Fig S5a). Once the cells entered the stationary phase, the consumption of 2-methylsuccinic acid was clearly reduced while the mesaconic acid decrease was almost completely prevented. Therefore, in addition to a higher maintenance of the product levels, the ratio between the two products changed in the course of the stationary phase.

### Investigation of transporter deletion effects on citramalic acid production

**Fig. 8:**
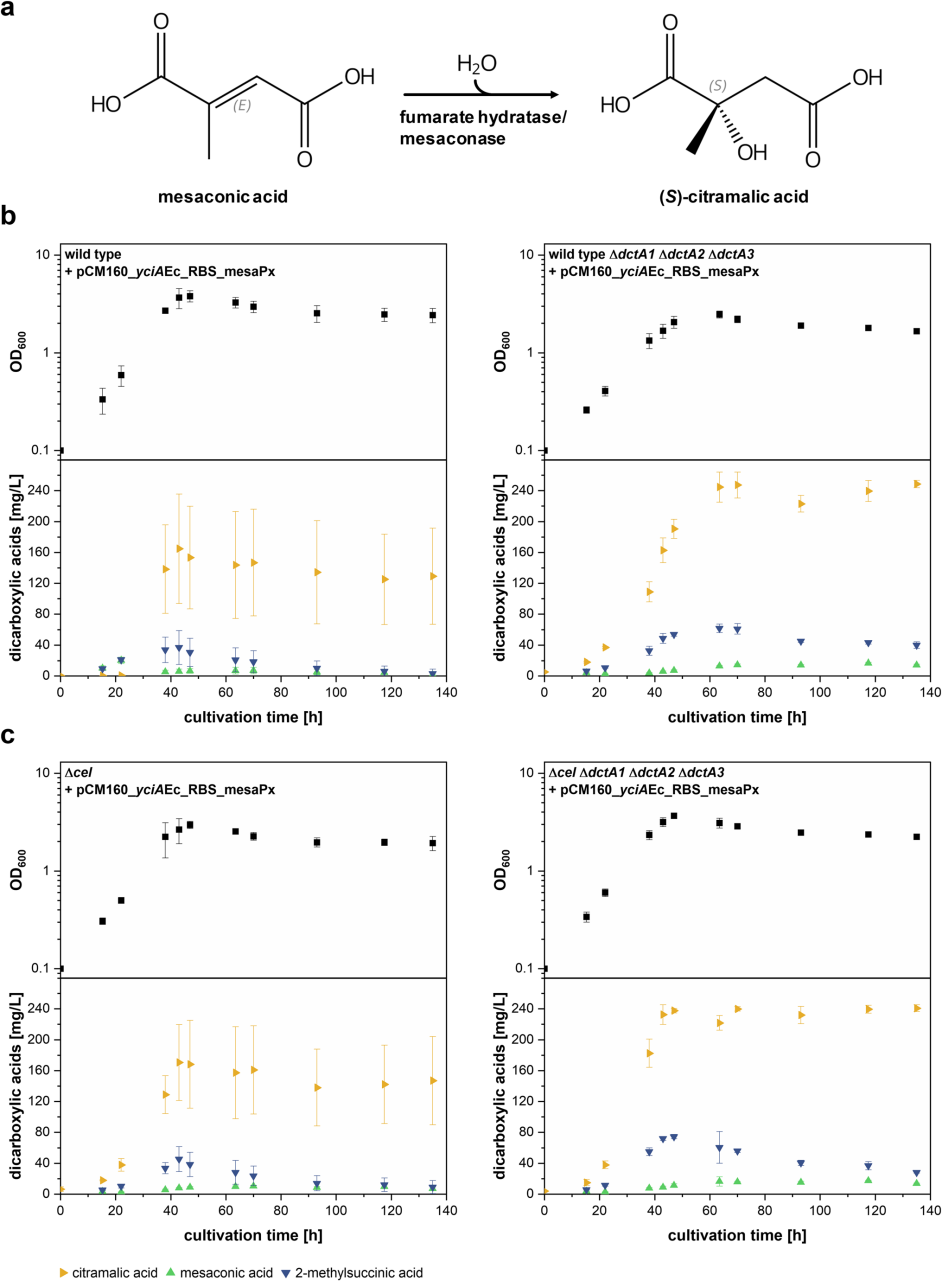
Production of citramalic acid with strains lacking DctA transporters. **a** Reaction scheme for hydration of mesaconic acid to (*S*)-citramalic acid catalyzed by mesaconase/fumarate hydratase (from Kronen et al. 2015). Absolute stereochemistry and double bond geometry are labeled in gray. **b** Growth kinetics and time-dependent concentration of mesaconic acid, 2-methylsuccinic acid and citramalic acid in supernatant of *M. extorquens* AM1 wild type and triple *dctA* transporter deletion strain expressing thioesterase encoding gene *yciA*HI and mesaconase in methanol minimal medium. **c** Growth kinetics and time-dependent concentration of mesaconic acid, 2-methylsuccinic acid and citramalic acid in supernatant of ∆*cel* and ∆*cel* triple *dctA* transporter deletion strain expressing thioesterase encoding gene *yciA*HI and mesaconase from *P. xenovorans* in methanol minimal medium cultures. Other EMCP-derived carboxylic acid products with titers insufficient for quantification (< 5 mg/L) are not displayed. Error bars represent standard deviations from three independent transformants. An additional visualization of the data in form of products per OD_600_ can be found in Online Resource Fig. S6

An alternative way to reduce reuptake of dicarboxylic acid products is by converting them to non-metabolizable products. By introduction of a mesaconase/fumarate hydratase from *P. xenovorans* (Kronen et al. 2015) in addition to YciAEc, we were able to convert mesaconic acid to citramalic acid by enzymatic hydration (Fig. 8a). Analytical evidence for the conversion is given in Online Resource Fig. S1. In contrast to mesaconic acid and 2-methylsuccinic acid, citramalic acid is most probably not taken up and metabolized by the cell, as its concentration stayed stable during the stationary phase (Fig. 8b,c). However, cell density values and product measurements of *M. extorquens* AM1 and *M. extorquens* AM1 ∆*cel* strains carrying pCM160_*yciA*Ec_RBS_mesaPx showed high standard deviations for the biological replicates. The maximal citramalic acid concentration measured was 164 ± 71 mg/L or 170 ± 49 mg/L, respectively (Fig. 8b,c, left graphs). Use of corresponding triple *dctA* deletion strains resulted in clearly higher citramalic acid concentrations of approximately 249 ± 5 mg/L or 241 ± 4 mg/L, respectively (Fig. 8b,c, right graphs). Moreover, in both strain backgrounds, the transporter gene deletions obviously reduced the variations observed within three independent transformants.

## Discussion

In this study, we focused on the reduction of product reuptake in *M. extorquens* AM1 strains producing dicarboxylic acids as 2-methylsuccinic acid and mesaconic acid. This will serve as a basis for the development of stable and more productive strains for the future production of dicarboxylic acids from methanol.

In the past, product reuptake in the later cultivation phases reduced the product yield (Sonntag et al. 2014, 2015). It was postulated that there are two possible modes for product uptake: 1) it may be triggered upon transition from exponential to stationary growth phase or 2) the product uptake takes place over the entire course of cultivation and is masked by the high production rates during the early cultivation stages (Sonntag et al. 2014). In the second case, the productivity in the exponential growth phase would be reduced by the presence of a futile cycle. By repressing the reimport of products, one could eliminate this cycle and thus achieve higher productivity.

To identify potential import factors, we performed transcriptome analysis of a strain harboring plasmid pCM160_RBS_*yciA*HI, encoding the thioesterase YciA from *Haemophilus influenzae*. However, we could not identify genes that were clearly upregulated in stationary growth phase compared to early sampling points in the exponential growth phase and which were annotated to encode for transporters. When specifically analyzing the time-dependent expression levels of the *dctA* homologs and *kgtP* (targets suggested by Sonntag and colleagues (2014)) and comparing them with a control strain, we found slightly increased levels only for *dctA1*. After concluding that the transcriptome analysis was no appropriate approach to identify relevant transport factors, we chose a straightforward mutant selection approach using a cytotoxic dicarboxylic acid.

In preliminary tests, DFS toxicity surprisingly applied only to methanol- and acetate-grown and not to succinate-grown cells. As its toxicity could furthermore be compensated by supplementing the cells with glyoxylate, we concluded that DFS toxicity is not only mediated by the EMCP but directly targets it. Since DFS has a high structural similarity to the dicarboxylic acids that constitute the EMCP intermediates in their CoA-activated form, DFS might act as competitive inhibitor. Glyoxylate supplementation has previously been shown to fully restore methylotrophic growth of Δ*ccr*, Δ*epi*, and Δ*ecm* strains and to partially restore growth of Δ*msd* strains (Schada von Borzyskowski et al. 2018), so we can speculate that DFS acts on one or several of the corresponding EMCP enzymes.

Using a simple selection approach, we were able to isolate DFS-resistant *M. extorquens* AM1 mutants from DFS-containing methanol minimal medium or acetate minimal medium. In one of the two mutants selected for further investigations, DFS resistance was accompanied by reduced uptake of mesaconic acid and 2-methylsuccinic acid. DFS mutant 2, in which we identified a mutation in a putative dicarboxylic acid transporter encoding gene (SLC13 family), was resistant to DFS. Nevertheless, the strain showed 2-methylsuccinic acid and mesaconic acid uptake behavior comparable to a non-mutated control strain. In contrast, mutation or deletion of *dctA2*, led to strong reduction of product reuptake. The corresponding mutant strain, DFS mutant 1, not only exhibited the DFS resistance phenotype we selected for, but in addition showed clearly reduced reuptake of mesaconic acid and 2-methylsuccinic acid. Since the strain was still able to grow on succinate, we assume that *dctA2* is not identical to the open reading frame designated as A33597 in Van Dien’s study (Van Dien et al. 2003).

Although we did not perform complementation experiments with dctA2, strong indications for a dicarboxylic acid uptake function of DctA2 exist. The fact that also a decreased sodium concentration resulted in a ceasing of dicarboxylic acid reuptake (Sonntag et al. 2015) is in line with this assumption, as DctA transporters are sodium-dependent (Janausch et al. 2002). The finding of identical acid uptake phenotypes with a *dctA2* deletion strain is an additional argument for DctA2 to be the causal factor for the observed reuptake defect. It furthermore demonstrated that the removal of four amino acids from the protein in the DFS mutant 1 probably results in a complete loss of function with respect to mesaconic acid and 2-methylsuccinic acid uptake. Possible explanations for the residual uptake activity observed for *dctA2* mutants were contributions of DctA2 homologs DctA1 and DctA3. After we found out that single deletions of *dctA1* or *dctA3* did not cause changes to the product uptake kinetic, we initially hypothesized that these factors might nevertheless be responsible for the residual uptake activity seen for the *dctA2* mutants. A conceivable scenario would be that transcription of their genes is activated when DctA2 is absent. However, we ruled out this possibility by testing mutant strains in which all three dctA genes were deleted. Only combinations of deletions that included the *dctA2* deletion resulted in clearly reduced product reuptake. The fact that product yields were maintained in stationary phase but that no product increase per biomass could be observed during growth for the *dctA2* mutants compared to the wild type (Fig. S5a), may suggest that uptake is likely to only occur if a threshold of product concentration is reached or carbon becomes limiting. Another aspect revealed from the analysis of the production kinetics of the *dctA2* mutant strains is based on the observation that the reduced reuptake does not apply to the two products in the same way. Expression of *yciA* from *H. influenzae* in *M. extorquens* AM1 wild type strain in our experiments usually resulted in release of similar amounts of the two dicarboxylic acid products, with a slightly higher level of 2-methylsuccinic acid. The *dctA2* deletion seems especially to prevent mesaconic acid uptake, while 2-methylsuccinic acid is still imported to a certain extent. This observation once more suggests the presence of other, yet unidentified transporters involved at least in 2-methylsuccinic acid uptake. The possibility of passive diffusion of completely protonated acids through the membrane is furthermore ruled out by the different kinetics of the two acids. As the two products have comparable pKa values and show high structural similarity, strong differences in membrane passing rates are not expected.

Besides prevention of product reuptake in the stationary phase, we also aimed at elimination of product uptake potentially taking place in the production phase during exponential growth. In the experiments conducted, the mesaconic acid and 2-methylsuccinic acid production kinetics in the exponential growth phase was the same, both with and without *dctA2* deletion. Although the overall product titer was not increased, the process is much more robust when performed in a *dctA2* deletion strain as the harvest time for achieving maximum product yield is no longer as time critical.

For the production of citramalic acid, however, the use of the *dctA* triple deletion strain resulted not only in a significant gain in robustness, but also in production yield. Citramalic acid as a non-natural product of *M. extorquens* AM1 is not reimported by the cells, as shown by the respective experiment with the control strain. Although a clear explanation for the improvements of citramalic acid production in the DctA mutant strain compared to the control strain cannot be drawn from the experiments, reduced mesaconic acid uptake is probably causal for the observed phenotypes. This reduced uptake might slow down its further metabolization and thereby increase the carbon flux towards citramalic acid. The use of a *dctA* triple deletion strain for citramalic production led to a 1.4-fold increase in mean product yield and a substantial reduction of the standard deviations. The study that first described mesaconase/fumarate hydratase from *P. xenovorans* demonstrated its enantioselectivity towards (*S*)-citramalic acid (Kronen et al. 2015). We therefore assumed that the constructed *M. extorquens* AM1 strains also produce enantiopure (*S*)-citramalic acid.

Although we were not able to achieve higher overall yields for mesaconic acid or 2-methylsuccinic acid, the use of a *dctA2* deletion strain almost completely prevented the reuptake of mesaconic acid and part of 2-methylsuccinic acid within the stationary growth phase. Furthermore, the product yield of citramalic acid was significantly increased in the *dctA2* deletion strain. Therefore, the dicarboxylic acid transporter deletion mutants provided here are excellent starting points for the creation of different dicarboxylic acid production strains.

## Supplementary Information

Below is the link to the electronic supplementary material.

Supplementary file1 (PDF 955 KB)

## Data Availability

All data generated or analyzed during this study are included in this published article and its supplementary information file.
