# Supplementary material for: Improvement of dicarboxylic acid production with Methylorubrum extorquens by reduction of product reuptake
Source: Appl Microbiol Biotechnol. 2022 Sep 15;106(19-20):6713–31. doi: 10.1007/s00253-022-12161-0 (PMC9529712; doi:10.1007/s00253-022-12161-0)
Supplement: Supplementary file 1 — Supplementary file1 (PDF 955 KB) [file 253_2022_12161_MOESM1_ESM.pdf]

Supplementary Information

Journal: Applied Microbiology and Biotechnology

Improvement of dicarboxylic acid production with *Methylorubrum extorquens* by reducing the product reuptake

Laura Pöschel[1], Elisabeth Gehr[1], Markus Buchhaupt[1]*

[1] DECHEMA-Forschungsinstitut, Microbial Biotechnology, Theodor-Heuss-Allee 25, 60486 Frankfurt am Main, Germany

[2] Department of Life Sciences of the Goethe University Frankfurt am Main, Max-von-Laue-Str. 9, 60438 Frankfurt am Main, Germany

*Corresponding author, Phone: +49-69-7564-629, e-mail address: markus.buchhaupt@dechema.de

Electronic supplementary material as Online Resource

Table S1 Details of genomic sequencing and mapping procedure

Table S2 Genetic differences between the sequenced strains and the used reference genome

Fig. S1 Analytical evidence for production of citramalic acid by an *M. extorquens* AM1 strain

Fig. S2 Dicarboxylic acid production per OD_{600} (transformed data from figure 2)

Fig. S3 Dicarboxylic acid production per OD_{600} (transformed data from figure 5)

Fig. S4 Dicarboxylic acid production per OD_{600} (transformed data from figure 6)

Fig. S5 Dicarboxylic acid production per OD_{600} (transformed data from figure 7)

Fig. S6 Dicarboxylic acid production per OD_{600} (transformed data from figure 8)

Table S1 Details of genomic sequencing and mapping procedure

Strain	Genetic entity	Total number of mapped Illumina reads	Base coverage of reference sequence	Average base coverage [mean ± standard deviation]
***M. extorquens* AM1 wild type**	NC_012807	317 955	100 %	514.6 ± 186.0
	NC_012808	17 352 981	99.998 %	225.9 ± 212.4
	NC_012809	235 559	100 %	445.3 ± 141.6
	NC_012810	180 717	100 %	519.8 ± 180.6
	NC_012811	3 828 584	99.99 %	216.9 ± 70.7
***M. extorquens* AM1 DFS mutant 1**	NC_012807	353 562	100 %	1061.5 ± 258.9
	NC_012808	18 846 841	100 %	452.3 ± 86.2
	NC_012809	216 117	100 %	764.7 ± 110.5
	NC_012810	139 576	100 %	750.8 ± 101.3
	NC_012811	4 279 978	99.9998 %	447.9 ± 76.9
***M. extorquens* AM1 DFS mutant 2**	NC_012807	295 048	100 %	869.9 ± 195.3
	NC_012808	15 563 801	100 %	367.0 ± 83.7
	NC_012809	166 635	100 %	580.1 ± 73.9
	NC_012810	104 196	100 %	550.5 ± 76.2
	NC_012811	3 425 166	99.997 %	352.7 ± 58.6

Table S2 Genetic differences between the sequenced strains and the used reference genome. The listed mutations were found in all sequenced strains including *M. extorquens* AM1 wild type. N/A indicates that there is no annotated gene function (annotation date 04/11/2021)

Genetic entity	Locus tag	Gene	Polymorphism type	Change	Effect on open reading frame
NC_012808	MEXAM1_RS02205	N/A	Insertion	(C)3 → (C)4	Frame Shift
NC_012808	MEXAM1_RS10680	N/A	Deletion	(G)3 → (G)2	Frame Shift
NC_012808	MEXAM1_RS34015	N/A	SNP (transversion)	T → G	None
NC_012808	MEXAM1_RS12610	N/A	SNP (transition)	C → T	None
NC_012808	MEXAM1_RS12610	N/A	SNP (transition)	T → C	None
NC_012808	MEXAM1_RS13695	treS	Deletion	(C)4 → (C)3	Frame Shift
NC_012808	MEXAM1_RS14240	N/A	Deletion	(G)3 → (G)2	Frame Shift
NC_012808	MEXAM1_RS32900	N/A	Deletion	-TGCCG	Frame Shift
NC_012808	Intergenic region	N/A	SNP (transition)	A → G	None
NC_012811	MEXAM1_RS28080	N/A	Insertion	+C	Frame Shift
NC_012811	MEXAM1_RS29025	N/A	Deletion	(G)3 → (G)2	Frame Shift

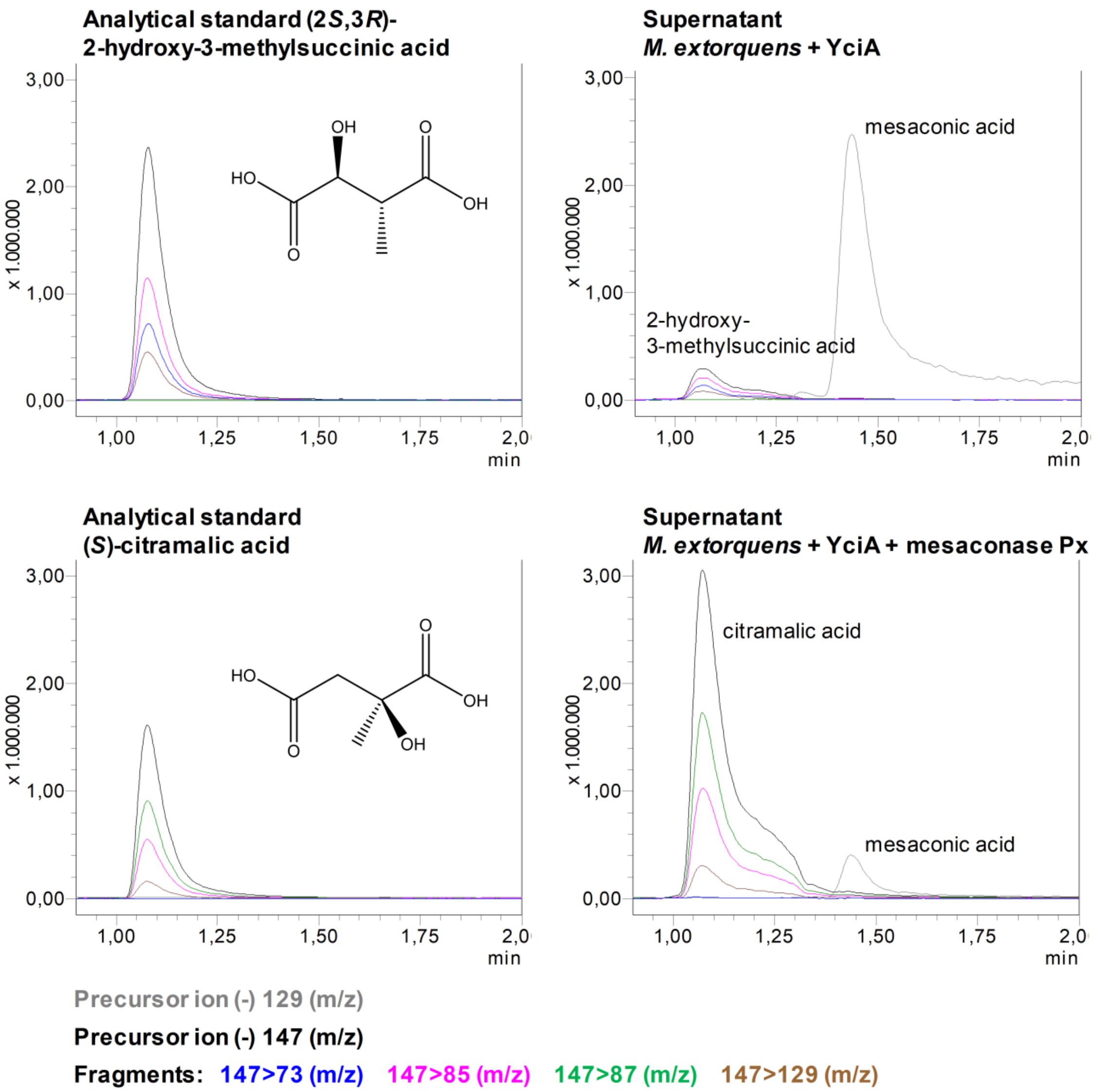

Fig. S1 Analytical evidence for production of citramalic acid by an *M. extorquens* AM1 strain expressing a mesaconase from *Paraburkholderia xenovorans* (mesaconase_Px) in addition to YciA from *Haemophilus influenzae*. Shown are chromatograms of precursor 129 m/z (mesaconic acid) and precursor 147 m/z (2-hydroxy-3-methylsuccinic acid or citramalic acid). To distinguish between 2-hydroxy-3-methylsuccinic acid (present at low concentrations in *M. extorquens* AM1 cultures) and citramalic acid, the analytes were fragmented using the identical LC-MS/MS MRM method detecting fragments 73, 85, 129 and 87. Here, the fragment of 87 (m/z) is characteristic for citramalic acid and can therefore be used for unambiguous identification. In supernatant of cultures with *M. extorquens* AM1 expressing thioesterase gene *yciA*, mesaconic acid as well as 2-hydroxy-3-methylsuccinic acid can be detected. With the additional introduction of a mesaconase, the mesaconase peak becomes smaller and conversion to citramalic acid can be observed.

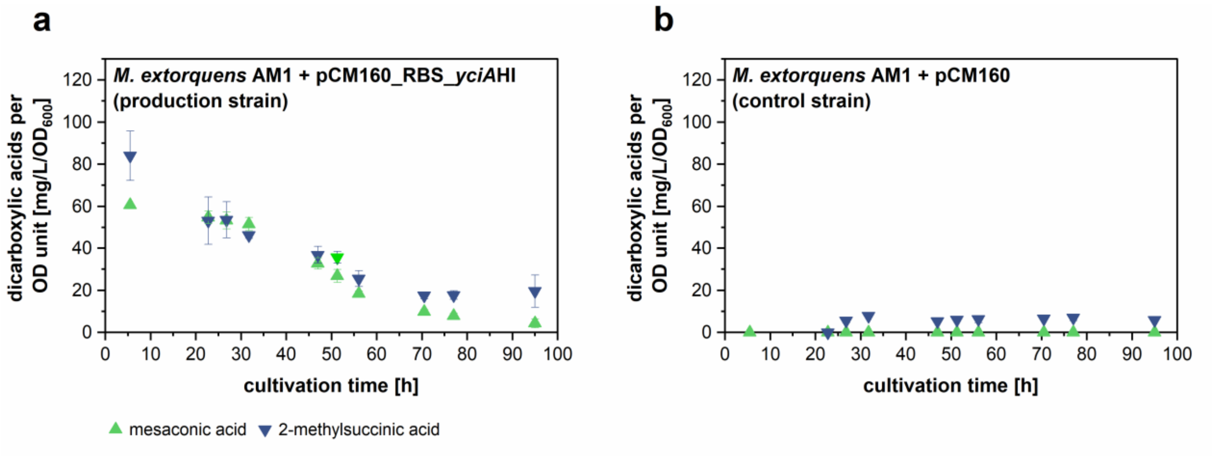

Fig. S2 Dicarboxylic acid production per OD_{600} (transformed data from figure 2). Mesaconic acid and 2-methylsuccinic acid concentrations per OD_{600} in supernatant of *M. extorquens* AM1 + pCM160_RBS_*yciAHI* (**a**) or *M. extorquens* AM1 + pCM160 (**b**) growing in methanol minimal medium. Error bars represent standard deviations from three independent replicates

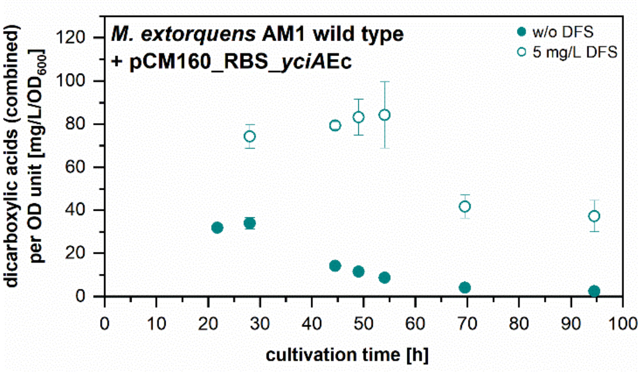

Fig. S3 Dicarboxylic acid production per OD_{600} (transformed data from figure 5). Mesaconic acid and 2-methylsuccinic acid concentrations (combined) per OD_{600} in supernatant of *M. extorquens* AM1 harboring pCM160_RBS_*yciA*Ec in methanol minimal medium (*filled symbols*) and in methanol minimal medium with addition of 5 mg/L 2,2-difluorosuccinic acid (DFS) after 5 h of cultivation (*empty symbols*). Error bars represent standard deviations from two independent replicates

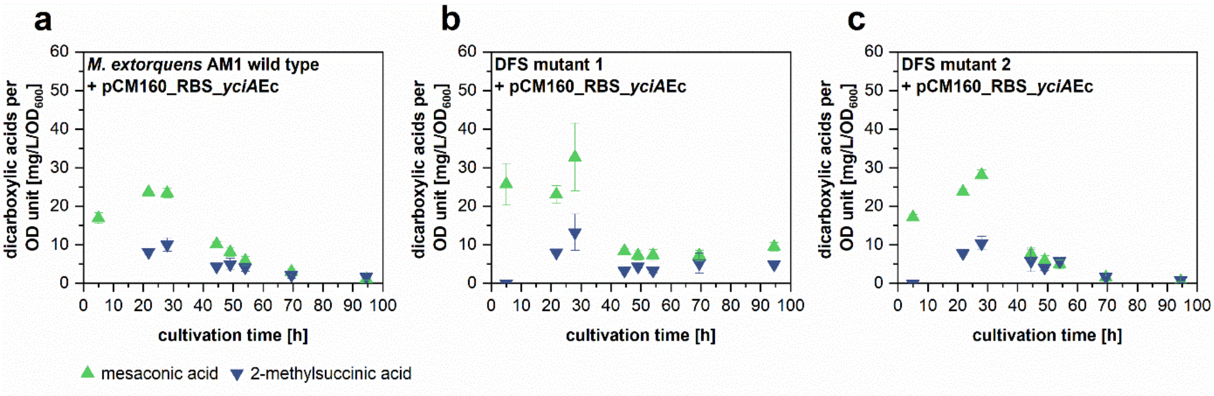

Fig. S4 Dicarboxylic acid production per OD600 (transformed data from figure 6). Mesaconic acid and 2-methylsuccinic acid concentrations per OD600 in supernatant of *M. extorquens* AM1 wild type + pCM160_RBS_*yciA*Ec (**a**), DFS mutant 1 + pCM160_RBS_*yciA*Ec (**b**) and DFS mutant 2 + pCM160_RBS_*yciA*Ec (**c**), growing in methanol minimal medium. Error bars represent standard deviations from three independent replicates

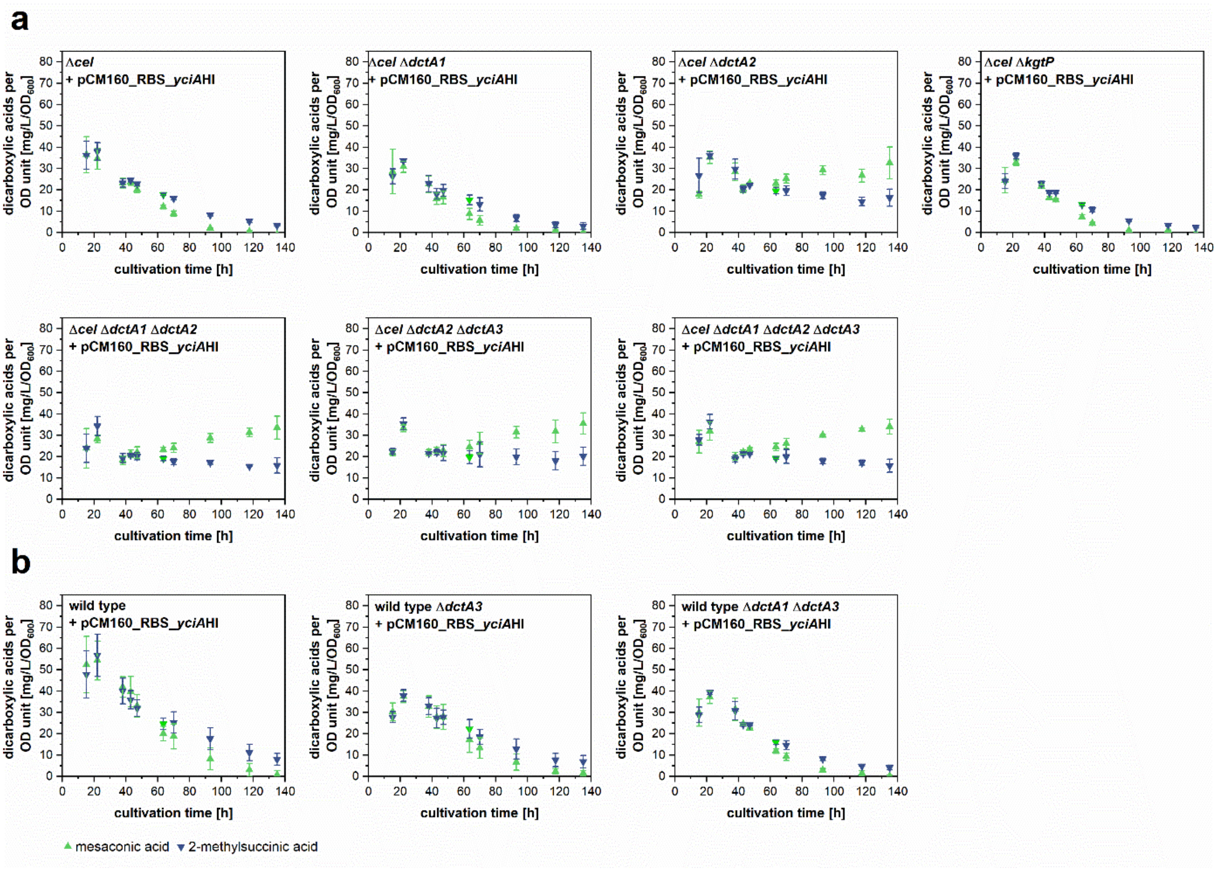

Fig. S5 Dicarboxylic acid production per OD600 (transformed data from figure 7). Mesaconic acid and 2-methylsuccinic acid concentrations per OD600 in supernatant of *M. extorquens* AM1 cells without and with single, double or triple transporter deletions. Strains heterologously express the thioesterase encoding gene *yciA*HI in methanol minimal medium. Error bars represent standard deviations from three independent replicates. Strains were constructed based on either **a** *M. extorquens* AM1 wild type or **b** *M. extorquens* AM1 Δ*cel* strain

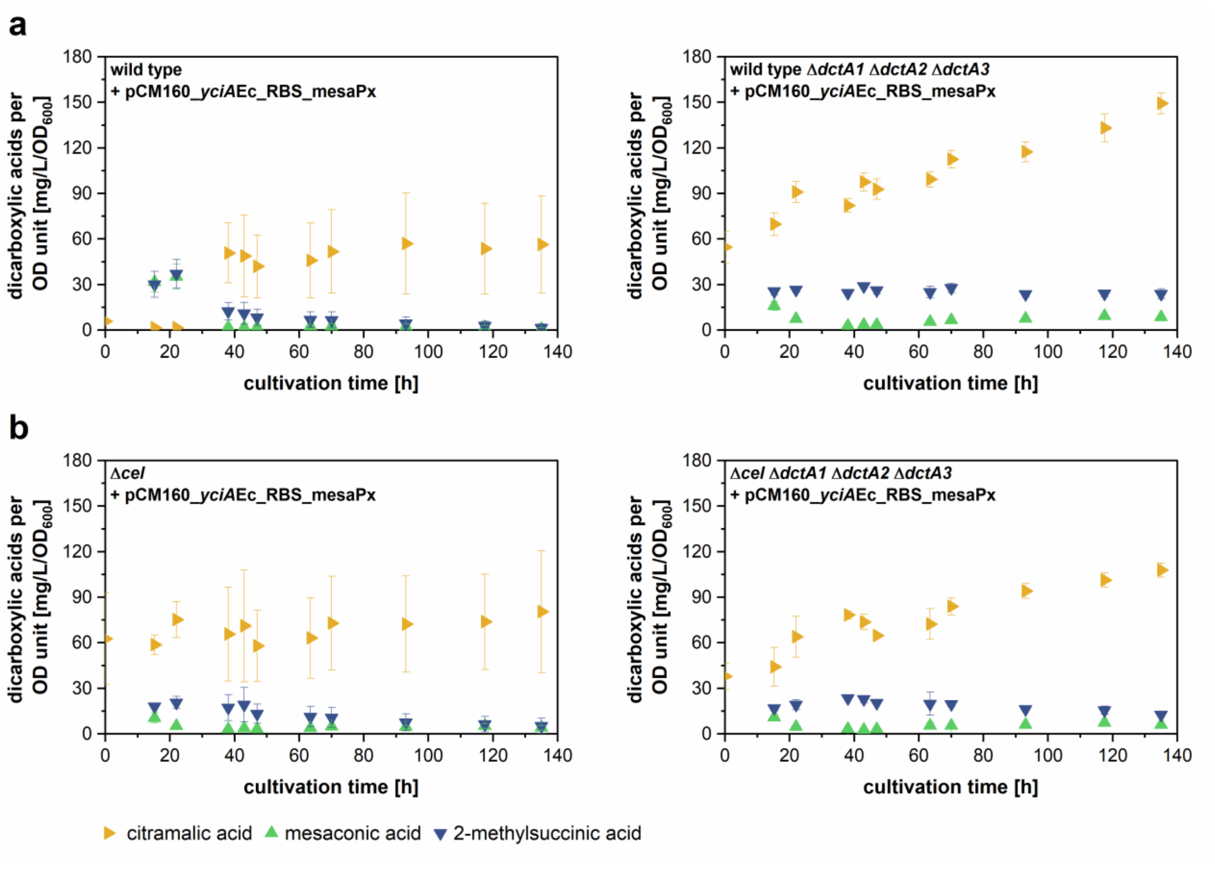

Fig. S6 Dicarboxylic acid production per OD_{600} (transformed data from figure 8). Mesaconic acid, 2-methylsuccinic acid and citramalic acid concentrations per OD_{600} in supernatant of *M. extorquens* AM1 wild type and triple *dctA* transporter deletion strain expressing thioesterase encoding gene *yciA*HI and mesaconase in methanol minimal medium
